# Serotonin 2C Antagonism in the Lateral Orbitofrontal Cortex Ameliorates Cue-Enhanced Risk Preference and Restores Sensitivity to Reinforcer Devaluation in Male Rats

**DOI:** 10.1523/ENEURO.0341-21.2021

**Published:** 2021-12-07

**Authors:** Brett A. Hathaway, Jackson D. Schumacher, Kelly M. Hrelja, Catharine A. Winstanley

**Affiliations:** Department of Psychology, Djavad Mowafaghian Centre for Brain Health, University of British Columbia, Vancouver, British Columbia V6T 1Z3, Canada

**Keywords:** decision making, flexibility, orbitofrontal cortex, reward, risk, serotonin

## Abstract

Previous research has indicated that reward-paired cues can enhance disadvantageous risky choice in both humans and rodents. Systemic administration of a serotonin 2C receptor antagonist can attenuate this cue-induced risk preference in rats. However, the neurocognitive mechanisms mediating this effect are currently unknown. We therefore assessed whether the serotonin 2C receptor antagonist RS 102221 is able to attenuate cue-enhanced risk preference via its actions in the lateral orbitofrontal cortex (lOFC) or prelimbic (PrL) area of the medial prefrontal cortex (mPFC). A total of 32 male Long–Evans rats were trained on the cued version of the rat gambling task (rGT), a rodent analog of the human Iowa gambling task, and bilateral guide cannulae were implanted into the lOFC or PrL. Intra-lOFC infusions of the 5-HT_2C_ antagonist RS 102221 reduced risky choice in animals that showed a preference for the risky options of the rGT at baseline. This effect was not observed in optimal decision-makers, nor those that received infusions targeting the PrL. Given prior data showing that 5-HT_2C_ antagonists also improve reversal learning through the same neural locus, we hypothesized that reward-concurrent cues may amplify risky decision-making through cognitive inflexibility. We therefore devalued the sugar pellet rewards used in the cued rGT (crGT) through satiation and observed that decision-making patterns did not shift unless animals also received intra-lOFC RS 102221. Collectively, these data suggest that the lOFC is one critical site through which reward-concurrent cues promote risky choice patterns that are insensitive to reinforcer devaluation, and that 5-HT_2C_ antagonism may optimize choice by facilitating exploration.

## Significance Statement

Lights and sounds signaling reward are used extensively in electronic gaming machines. Recent data indicate that these win-associated cues can increase disadvantageous risky choice. Administering a serotonin 2C receptor antagonist can ameliorate this effect in rats, potentially by increasing flexibility in decision-making. The orbitofrontal cortex (OFC) is critically involved in mediating flexible behavior. Thus, the present study evaluated whether serotonin 2C antagonism in the OFC can reduce disadvantageous risky choice via alterations to behavioral flexibility. Results implicate the OFC as one critical locus, and an increase in flexibility as a potential cognitive mechanism, through which cue-enhanced risky decision-making may improve. This could point to potential therapeutic interventions for problematic gambling that target the control of cues over behavior.

## Introduction

Win-associated audiovisual cues are used extensively in electronic games, smartphone apps, and commercial gambling products. Although these cues seem harmless, this form of sensory enhancement can impair decision-making. Specifically, adding win-concurrent cues to laboratory-based gambling tasks increases risky choice in rats and humans (Barrus and Winstanley, 2016; Cherkasova et al., 2018). Risky decision-making can contribute to the onset and maintenance of addiction disorders (Bolla et al., 2003; Goudriaan et al., 2005; Stevens et al., 2013). As such, the ability of reward-synchronous cues to increase risky choice may facilitate the development of pathologic gambling. Determining the neural and cognitive basis of this effect may shed light on how electronic games can become addictive and identify potential therapeutic interventions.

In rats, we have studied cue-driven risky choice using the rat gambling task (rGT), loosely analogous to the Iowa gambling task used clinically (Bechara et al., 1994; Zeeb et al., 2009). In both tasks, maximal reward is attained by avoiding the high-risk, high-reward options and instead favoring the options associated with lower per-trial gains. On the rGT, these low-risk, low-reward options result in less frequent and shorter time-out penalties and therefore more sugar pellets are earned overall. The addition of reward-paired audiovisual cues leads to greater risky choice on average (Barrus and Winstanley, 2016). Decision-making on the cued rGT (crGT) is subject to unique pharmacological regulation. Systemic administration of a serotonin (5-HT) 2C receptor antagonist SB242084 decreased risky choice selectively on the crGT while increasing premature responding, a reliable index of motor impulsivity, on both the cued and uncued versions of the task (Adams et al., 2017).

This finding was somewhat unexpected, given the previously described role of the 5-HT_2C_ receptor in cue-mediated behaviors such as cue-induced reinstatement of cocaine seeking and responding for a conditioned reinforcer (Pentkowski et al., 2010; Browne et al., 2017). Modulation of both mesolimbic dopamine release and activity within the medial prefrontal cortex (mPFC) were implicated in these results, and the findings were attributed to alterations in the incentive salience of the cues. Increased premature responding induced by 5-HT_2C_ antagonism on the rGT (Adams et al., 2017) and the 5-choice serial reaction time task (5CSRT; Winstanley et al., 2004) may also depend on the nucleus accumbens, as systemic administration enhances accumbal dopaminergic release (Browne et al., 2017), which can increase this form of impulsivity (Pattij et al., 2007; Economidou et al., 2012). Indeed, infusions of a 5-HT_2C_ antagonist into the accumbens of rats increased premature responding on the 5CSRT, whereas microinjections into the mPFC had little effect (Robinson et al., 2008).

These findings suggest that 5-HT_2C_ receptor antagonism in the nucleus accumbens would enhance the control of cues over behavior, and 5-HT_2C_ agonism would attenuate it. However, we instead found that systemic administration of the antagonist ameliorated the risk-enhancing effect of cues on the rGT, while the agonist was without effect. As such, 5-HT_2C_ antagonism may mitigate cue-driven risky choice through a different mechanism than described above, and distinct neural loci.

5-HT, particularly the 5-HT_2C_ receptor, is critically involved in mediating flexible behavior (Barlow et al., 2015). Administration of a 5-HT_2C_ antagonist into rats’ lateral orbitofrontal cortex (lOFC) but not the mPFC reduced perseveration during reversal learning (Boulougouris and Robbins, 2010). Several studies have identified the lOFC as a critical region for flexibility in decision-making (Baxter et al., 2000; Schoenbaum et al., 2002; Izquierdo et al., 2004; Amodeo et al., 2017). On the uncued rGT, the lOFC is involved in determining the optimal decision-making strategy (Zeeb and Winstanley, 2011). Interestingly, inactivating the lOFC during acquisition of the crGT may increase optimal choice (Ferland, J.-M. N., Barrus, M. M., Betts, G. D., and Winstanley, C. A., unpublished observations). As such, the inclusion of cues may impair decision-making by altering the establishment of accurate action-outcome contingencies in the lOFC. Theoretically, manipulating serotonergic activity in this region could reintroduce flexibility in the stored action-outcome contingencies and thereby ameliorate this effect.

We therefore administered the 5-HT_2C_ antagonist RS 102221 directly into the lOFC and assessed performance on the crGT. To confirm the regional specificity of any observed effects, we also targeted the prelimbic (PrL) region of the mPFC. We hypothesized that intra-lOFC, but not intra-PrL, RS 102221 would attenuate risky decision-making. We also tested whether decision-making on the crGT is less flexible compared with the uncued rGT by evaluating sensitivity to reinforcer devaluation. Finally, we investigated whether intra-lOFC RS 102221 could restore sensitivity to this manipulation, as expected if 5-HT_2C_ antagonism improves decision-making by reinstating flexibility.

## Materials and Methods

### Subjects

Subjects were 48 male Long–Evans rats (Charles River Laboratories) weighing 275–300 g on arrival to the facility. One to two weeks following arrival, rats were food-restricted to 14 g of rat chow per day and were maintained at least 85% body weight of an age-matched and sex-matched control (initial weight before food restriction: M = 353 g, SD = 51 g; weight before surgery: M = 399 g, SD* *=* *31 g). Water was available *ad libitum*. All subjects were pair-housed or trio-housed in a climate-controlled colony room under a 12/12 h reverse light/dark cycle (21°C; lights off at 8 A.M.). Huts and paper towel were provided as environmental enrichment. Behavioral testing took place 5 d per week. Housing and testing conditions were in accordance with the Canadian Council of Animal Care, and experimental protocols were approved by the UBC Animal Care Committee.

### Behavioral apparatus

Testing took place in 32 standard five-hole operant chambers, each of which was enclosed in a ventilated, sound-attenuating chamber (Med Associates Inc). Chambers were fitted with an array composed of five equidistantly spaced response holes. A stimulus light was located at the back of each hole, and nose-poke responses into these apertures were detected by vertical infrared beams. On the opposite wall, sucrose pellets (45 mg; Bioserv) were delivered to the magazine via an external pellet dispenser. The food magazine was also fitted with a tray light and infrared sensors to detect sucrose pellet collection. A house light could illuminate the chamber. The operant chambers were operated by software written in Med-PC by CAW, running on an IBM-compatible computer.

### crGT training and testing

Details of training and testing have been reported previously (Zeeb et al., 2009; Barrus and Winstanley, 2016). Rats were first habituated to the operant chambers in two daily 30-min sessions, during which sucrose pellets were present in the nose-poke apertures and food magazine. Rats were then trained on a variant of the 5CSRT (Carli et al., 1983), in which rats were required to make a nose-poke response in one of the four apertures, indicated by a 10 s stimulus light. A correct response was rewarded by delivery of one sugar pellet to the food magazine. The location of the stimulus light varied between holes 1, 2, 4, and 5 across the session. Sessions lasted 30 min and consisted of ∼100 trials. Rats were trained until they reached a criteria of ≥ 50 correct responses with ≥80% accuracy and ≤20% omissions. Rats were then trained on a forced-choice variant of the crGT for seven sessions, in which rats were presented with one of the four options per trial in a pseudo-random fashion. This ensured rats had equal exposure to each reinforcement contingency before training on the free-choice version of the program.

**Figure 1.**
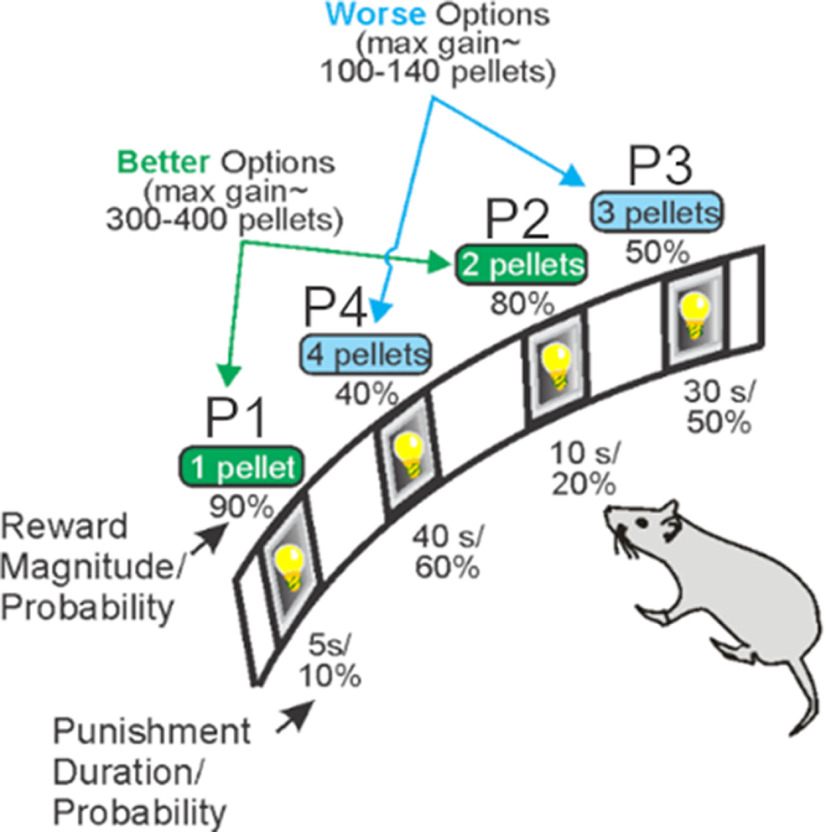
Task schematic of the crGT. A nose poke response in the food tray extinguished the traylight and initiated a new trial. After an ITI of 5 s, four stimulus lights were turned on in holes 1, 2, 4, and 5, each of which was associated with a different number of sugar pellets. The order of the options from left to right was counter-balanced within each cohort to avoid development of a simple side bias [version A (shown): P1, P4, P3, P2; version B: P4, P1, P3, P2]. The animal was required to respond at a hole within 10 s. This response was then rewarded or punished depending on the reinforcement schedule for that option. Reward delivery was accompanied by a 2-s audiovisual cue that increased in complexity with reward magnitude. If the animal lost, the stimulus light in the chosen hole flashed at a frequency of 0.5 Hz for the duration of the punishing time out, and all other lights were extinguished. The maximum number of pellets available per 30-min session shows that P1 and P2 are better than P3 and P4. The percent choice of the different options is one of the primary dependent variables. A score variable is also calculated, as for the IGT, to determine the overall level of risky choice as follows: [(P1 + P2) – (P3 + P4)]. Figure is modified from Barrus and Winstanley (2016).

A task schematic of the crGT is provided in Figure 1. During the 30-min session, trials were initiated by a nose-poke response within the illuminated food magazine. This response extinguished the light and started a 5-s intertrial interval (ITI). Any response at the five-hole array during the ITI was recorded as a premature response and punished by a 5-s time-out period, during which the house light was illuminated and no reward could be earned.

Following the ITI, apertures 1, 2, 4, and 5 in the five-hole array were illuminated for 10 s. If the rat failed to nose-poke in any illuminated hole within 10 s, the trial was recorded as an omission, the food magazine was re-illuminated, and rats were required to initiate a new trial. A nose-poke response within an illuminated aperture was either rewarded or punished according to that aperture’s reinforcement schedule. Probability of reward varied among options (0.9–0.4, P1–P4), as did reward size (one to four sucrose pellets). Punishments were signaled by the light within the chosen aperture flashing at a frequency of 0.5 Hz, which lasted for a 5- to 40-s time-out penalty depending on the aperture selected. Two-second compound tone/light cues occurred concurrently with reward delivery. Cue complexity and variability scaled with reward size. The task was designed such that the optimal strategy to earn the highest number of sucrose pellets during the 30-min session would be to exclusively select the P2 option, because of the relatively high probability of reward (0.8) and short, infrequent time-out penalties (10 s, 0.2 probability). While options P3 and P4 provide higher per-trial gains of three or four sucrose pellets, the longer and more frequent time-out penalties associated with these options greatly reduces the occurrence of rewarded trials, such that consistently selecting these options results in fewer sucrose pellets earned across the session and are therefore considered disadvantageous.

The position of each option for the crGT was counterbalanced across rats such that half the animals were trained on version A (left to right arrangement: P1, P4, P2, P3) and the other half on version B (left to right arrangement: P4, P1, P3, P2) to mitigate potential side bias. Rats received five training sessions per week.

### Surgery

When baseline performance was deemed statistically stable (following ∼40 training sessions), 32 animals were anesthetized with 2% isoflurane in O_2_ and 23-gauge stainless steel guide cannulae were implanted above the lOFC (*n *=* *20; AP = +3.5 mm, ML = ±2.6 mm from bregma, DV = −2.9 mm from dura) or the PrL (*n *=* *12; AP = +3.0 mm, ML = ±0.7 mm from bregma, DV = −2.8 mm from dura), using standard stereotaxic techniques. Guide cannulae were fixed to the skull via four stainless steel screws and dental acrylic, and obdurators flush with the end of the cannulae were inserted. Animals were given at least one week of recovery in their home cages before subsequent testing.

### Drug preparation

Three concentrations of the compound RS 102221 hydrochloride (Tocris Bioscience) were prepared each dosing day. First, 1 mg of the drug was suspended in 300 μl of 0.1 m HCl via sonication. The pH level was then adjusted to 6–7 with 1.0 and 0.1 m NaOH and saline to a final concentration of 2 mg/ml. Two aliquots of the solution were further diluted to 0.2 and 0.6 mg/ml. The highest concentration dose was vortexed before each infusion to prevent precipitation of the drug during the procedure. The vehicle solution consisted of saline that was pH-adjusted to 6–7 with NaOH.

### Microinfusion procedure

Following recovery, animals performed 10 free-choice sessions, after which all individuals displayed stable behavior. Animals were then habituated to the microinfusion process with two mock infusions, during which 30-gauge dummy injectors were inserted for 2 min but no infusion was performed, followed by a behavioral testing session initiated 10 min later. Infusions adhered to a 3-d cycle starting with a baseline session, followed by a drug or vehicle injection session, and then by a non-testing day; 0.5 μl per hemisphere injections of saline or RS 102221 (0.1, 0.3, or 1.0 μg of drug per hemisphere) were administered bilaterally at a rate of 0.3 μl/min with injectors that extended 0.8 mm beyond the guide cannulae. Injectors were left in place for an additional minute to allow for diffusion. Animals received each dose of RS 102221 plus vehicle, counterbalanced in a Latin Square design (for doses A thru D: ABCD, CADB, BDAC, DCBA). Once the microinfusions were completed, injectors were removed, obdurators replaced, and animals were placed in the operant chambers for 10 min before initiation of the crGT.

### Reinforcer devaluation

Twelve lOFC-cannulated rats and 16 surgically-naive rats underwent a reinforcer devaluation procedure. For the naive rats, this procedure took place across 2 d. On the first day, half of the rats were given *ad libitum* access to the sucrose pellets used as a reward on the crGT for 1 h before task initiation. The remaining rats completed the crGT without prior access to sucrose pellets. Following a baseline session day for which no sucrose pellets were administered before the task to any rats, the groups were then reversed and the other half were given 1-h access to sucrose pellets. To prevent the accumulation of damage of multiple infusions from impacting the results of this procedure, only one session of reinforcer devaluation was completed for cannulated rats. Ten minutes before task initiation, half of the rats received 1.0 μg of RS 102221 per hemisphere, according to the microinfusion procedure specified above. The other half received a vehicle dose. All rats in this group were given *ad libitum* access to sucrose pellets for 1 h before task initiation.

### Histology

Following completion of all behavioral testing, animals were anesthetized with isoflurane and euthanized by carbon dioxide exposure. Brains were extracted and fixed in 4% formaldehyde for at least 24 h, transferred to a 30% sucrose solution, and then frozen and cut via cryostat into 40-*μ*m coronal sections. These sections were stained with cresyl violet for visualization, and the projected locations of the injector tips protruding from the guide cannulae were mapped onto standard sections from Paxinos and Watson (1998).

### Behavioral measures and data analysis

All statistical analyses were completed using SPSS Statistics 27.0 software (SPSS/IBM). As per previous reports, the following rGT variables were analyzed: percentage choice of each option (number of times option chosen/total number of choices × 100), risk score (calculated as percent choice of [(P1 + P2) − (P3 + P4)]), percentage of premature responses (number of premature responses/total number of trials initiated × 100), sum of omitted responses, sum of trials completed, and average latencies to choose an option and collect reward. Variables that were expressed as a percentage were subjected to an arcsine transformation to limit the effect of an artificially imposed ceiling (i.e., 100%). A statistically stable baseline was determined by a repeated-measures ANOVA across data from four consecutive sessions before surgery, following ∼40 training sessions, in which both the session factor and session × choice interaction were not significant. Animals with a mean positive baseline risk score were designated as “optimal,” whereas rats with negative risk scores were classified as “risk-preferring.”

Choice data were analyzed with a two-way repeated measures ANOVA with dose (four levels: vehicle, 0.1 μg, 0.3 μg, and 1.0 μg) and choice (four levels: P1, P2, P3, and P4) as within-subject factors. For all other variables, dose was the only within-subjects factor. Risk status (two levels: optimal, risk-preferring) was included as a between-subjects factor for all statistical analyses. For the analysis of the reinforcer devaluation data, devaluation (two levels: baseline, devaluation) and choice (four levels: P1–P4) were the within-subject factors and group (three levels: surgically-naive, vehicle, drug) and risk status were the between-subjects factors. The baseline session used for each group was as follows: naive rats, session without experimental manipulation (i.e., no devaluation); vehicle rats, vehicle data from Latin Square dosing regimen; drug rats, highest concentration dose data from Latin Square dosing regimen. In isolated cases where data were missing because of technical issues, mean replacements were used.

For all analyses, if sphericity was violated as determined by Mauchley’s test, a Huynh–Feldt correction was applied, and corrected *p* values’ degrees of freedom were rounded to the nearest integer. Results were deemed to be significant if *p* values were less than or equal to an α of .05. Any main effects or interactions of significance were further analyzed via *post hoc* one-way ANOVA or paired samples *t* tests with a Bonferroni correction applied for the number of comparisons made. Any *p* > 0.05 but *p* < 0.09 were reported as a statistical trend.

## Results

### Cannulae placements

**Figure 2.**
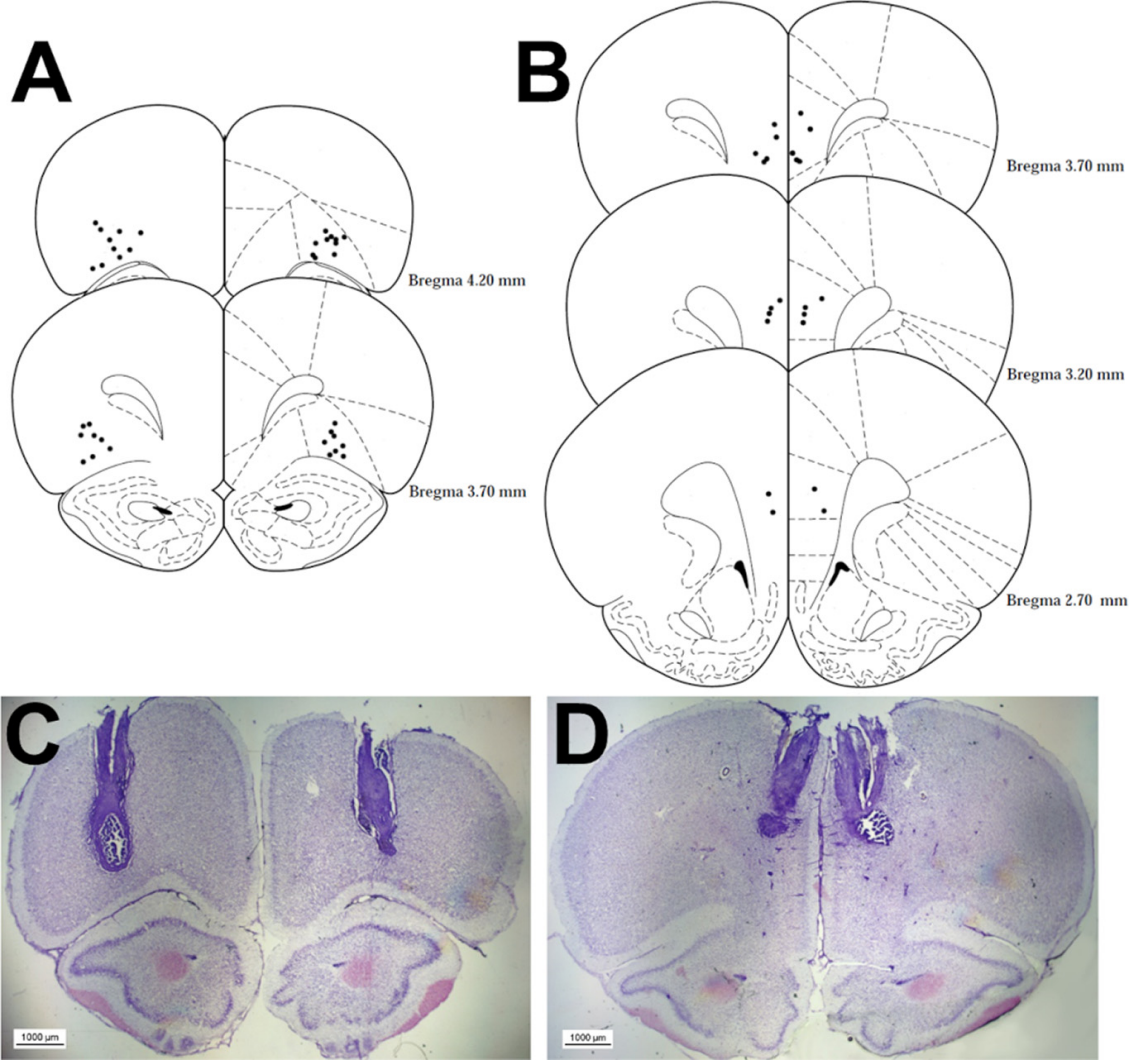
Histologic analysis of cannulae implantation. Location of all acceptable lOFC (***A***) and PrL region (***B***) infusions and an example microscopy image for each region in ***C***, ***D*** are shown. Coordinates are relative to bregma. Plates modified from Paxinos and Watson (1998).

The locations of all acceptable placements are depicted in Figure 2*A* for the lOFC cohort and Figure 2*B* for the PrL cohort. One animal in the lOFC condition did not survive surgery. One rat was excluded from the lOFC analyses because of inaccurate placement of the cannulae. All PrL cannulae placements were acceptable. This left a total of 18 (*n *=* *9 risk-preferring; *n *=* *9 optimal) and 12 (*n *=* *6 risk-preferring; *n *=* *6 optimal) rats for the lOFC and PrL analyses, respectively.

### Baseline behavior

One rat was excluded from the PrL condition because of poor task performance (mean of 30 trials completed per session, >30 s reward collection latency). As expected, risk-preferring rats selected the risky options at a significantly higher proportion than those who developed an optimal decision-making strategy for all baseline and saline sessions (choice × risk preference: *F*_(2,148)_ = 41.77, *p *<* *0.0001; optimal vs risk-preferring: P1: *t*_(75)_ = 1.60, *p *=* *0.11; P2: *t*_(75)_ = 12.89, *p *<* *0.0001; P3: *t*_(68)_ = −5.95, *p *<* *0.0001; P4: *t*_(66)_ = −3.97, *p *=* *0.0002). Across both cohorts, risk-preferring rats completed significantly fewer trials (risk preference: *F*_(1,25)_ = 38.43, *p *<* *0.0001), had a significantly higher proportion of premature responses (risk preference: *F*_(1,25)_ = 6.76, *p *=* *0.02), and exhibited shorter latencies to collect reward (risk preference: *F*_(1,25)_ = 14.67, *p *=* *0.001).

**Figure 3.**
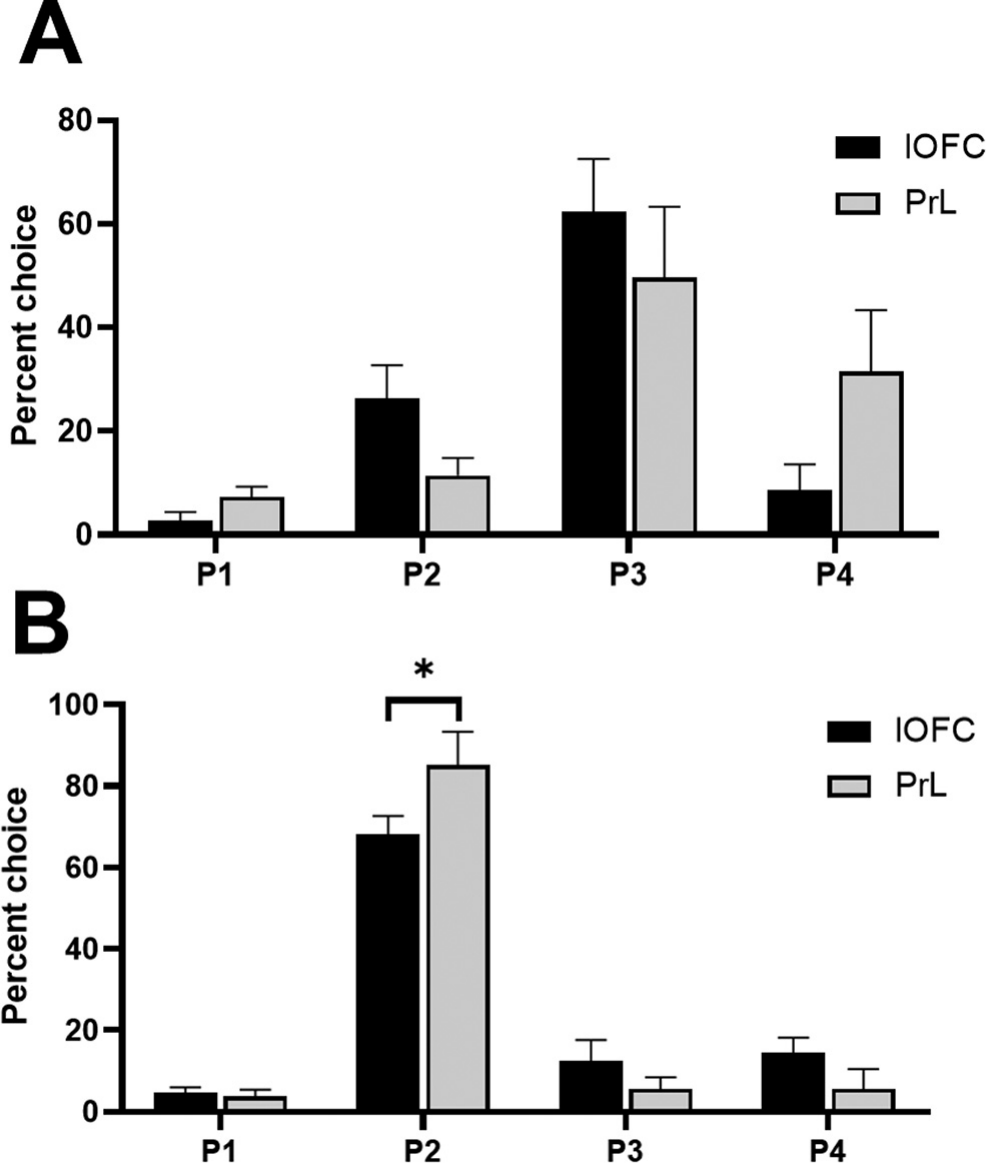
Baseline preferences for each option in the lOFC and PrL cohorts in risk-preferring rats (***A***) and optimal decision-makers (***B***). Optimal rats in the PrL cohort selected P2 at a significantly higher rate than optimal rats in the lOFC cohort. Data are expressed as mean ± SEM; *p < 0.05 according to an independent-groups *t* test.

The risk-preferring and optimal rats in the lOFC and PrL cohorts exhibited slightly different choice patterns (choice × brain region × risk preference: *F*_(2,51)_ = 5.11, *p *=* *0.009). This was particularly evident in optimal rats; risk-preferring rats exhibited only a trending difference in choice preference [risk-preferring rats: choice × brain region: *F*_(2,17)_ = 3.45, *p *=* *0.07 (Fig. 3*A*); optimal rats: choice × brain region: *F*_(3,38)_ = 3.99, *p *=* *0.02 (Fig. 3*B*)]. Optimal rats in the PrL group chose P2 at a significantly higher rate than rats in the lOFC group (*t*_(12)_ = 2.92, *p *=* *0.01).

To assess whether damage associated with cannula implantation and the microinfusion procedure impacted decision-making on the rGT, four presurgery sessions were binned and compared with baseline data collected between infusion days. No effect was observed on P1–P4 choice, indicating that procedure-associated damage did not significantly impact their decision-making (data bin × choice: *F*_(3,66)_ = 0.40, *p *=* *0.75).

### Microinfusions into lOFC

#### Choice

**Figure 4.**
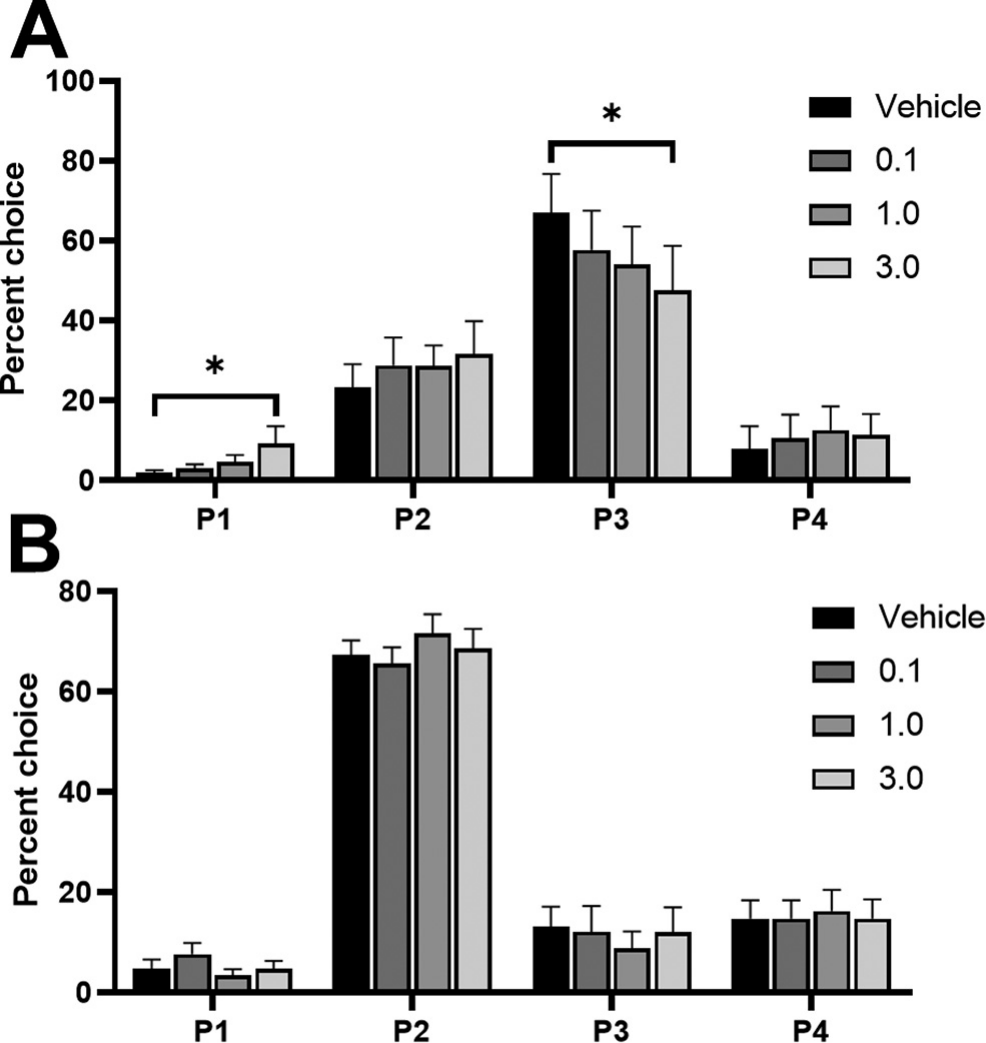
Effects of intra-lOFC infusions of the 5-HT_2C_ receptor agonist RS 102221 in (***A***) risk-preferring animals (*n *=* *9) and (***B***) optimal decision-makers (*n *=* *9) on P1–P4 choice. Antagonism of the 5-HT_2C_ receptor in the lOFC decreased P3 choice and increased P1 choice on the crGT in risk-preferring rats. Data are expressed as mean ± SEM; *p < 0.05 compared with vehicle.

We observed a significant shift in choice when comparing all doses in an omnibus ANOVA, that was dependent on the rats’ choice patterns at baseline (dose × choice × risk status: *F*_(7,117)_ = 7.34, *p *=* *0.04). This effect was only present in risk-preferring rats [dose × choice, risk-preferring: *F*_(9,72)_ = 3.09, *p *=* *0.003 (Fig. 4*A*); optimal: *F*_(9,72)_ = 0.01, *p *=* *0.30 (Fig. 4*B*)]. *Post hoc* analyses revealed a significant reduction in P3 choice (*t*_(8)_ = 2.49, *p *=* *0.04) and a significant increase in P1 choice (*t*_(8)_ = −2.74, *p *=* *0.03) in risk-preferring rats, when comparing vehicle to the highest dose. This resulted in a significant increase in risk score in these rats (dose: *F*_(3,24)_ = 4.39, *p *=* *0.01; vehicle vs 1.0 μg: *t*_(8)_ = −2.53, *p *=* *0.04). No effect was observed on P2 or P4 choice (P2: *t*_(8)_ = −1.52, *p *=* *0.17; P4: *t*_(8)_ = −1.59, *p *=* *0.15).

### Premature responding and other variables

No effect on premature responding was observed (dose: *F*_(3,48)_ = 1.10, *p *=* *0.36). A significant shift in omissions was evident in optimal rats only (dose × risk status: *F*_(3,38)_ = 3.66, *p *=* *0.03; dose, optimal: *F*_(3,21)_ = 3.56, *p *= 0.03; risk-preferring: *F*_(1,11)_ = 1.20, *p *=* *0.32). *Post hoc* analyses revealed a significant reduction in omitted trials in these rats, when comparing vehicle to the highest dose (*t*_(8)_ = 2.40, *p *=* *0.04). However, these rats showed a significantly higher level of omissions at vehicle compared with their baseline data (*t*_(8)_ = 2.41, *p *=* *0.04). No effect was observed on latency variables or trials completed (all *F *<* *1.33; *p *>* *0.27; Table 1).

**Table 1:** Mean values of all other task variables (% premature response, choice latency, collect latency, omissions, trials completed) at each dose of RS 102221 delivered into either the lOFC or PrL

Region	Dose	% Premature responses	Choice latency	Collect latency	Omissions	Trials completed
lOFC	0	28.62 ± 4.05	1.28 ± 0.19	1.32 ± 0.07	1.50 ± 0.53	75.38 ± 5.28
	0.1	29.66 ± 3.65	1.20 ± 0.12	1.19 ± 0.04	0.94 ± 0.22	77.56 ± 5.11
	1.0	31.90 ± 4.71	1.22 ± 0.12	1.24 ± 0.03	0.39 ± 0.18	75.63 ± 5.05
	3.0	25.92 ± 3.72	1.30 ± 0.13	2.01 ± 0.42	0.94 ± 0.51	83.63 ± 6.41
PrL	0	34.05 ± 5.35	1.13 ± 0.12	1.42 ± 0.16	1.36 ± 0.72	69.98 ± 7.94
	0.1	31.94 ± 6.59	1.14 ± 0.12	1.29 ± 0.05	0.91 ± 0.44	73.37 ± 9.67
	1.0	28.64 ± 6.30	1.31 ± 0.17	1.21 ± 0.07	1.18 ± 0.46	75.41 ± 10.12
	3.0	24.19 ± 5.00	1.34 ± 0.15	1.24 ± 0.12	0.91 ± 0.44	78.66 ± 10.78

Data are mean ± SEM.

### Reinforcer devaluation

#### Choice

**Figure 5.**
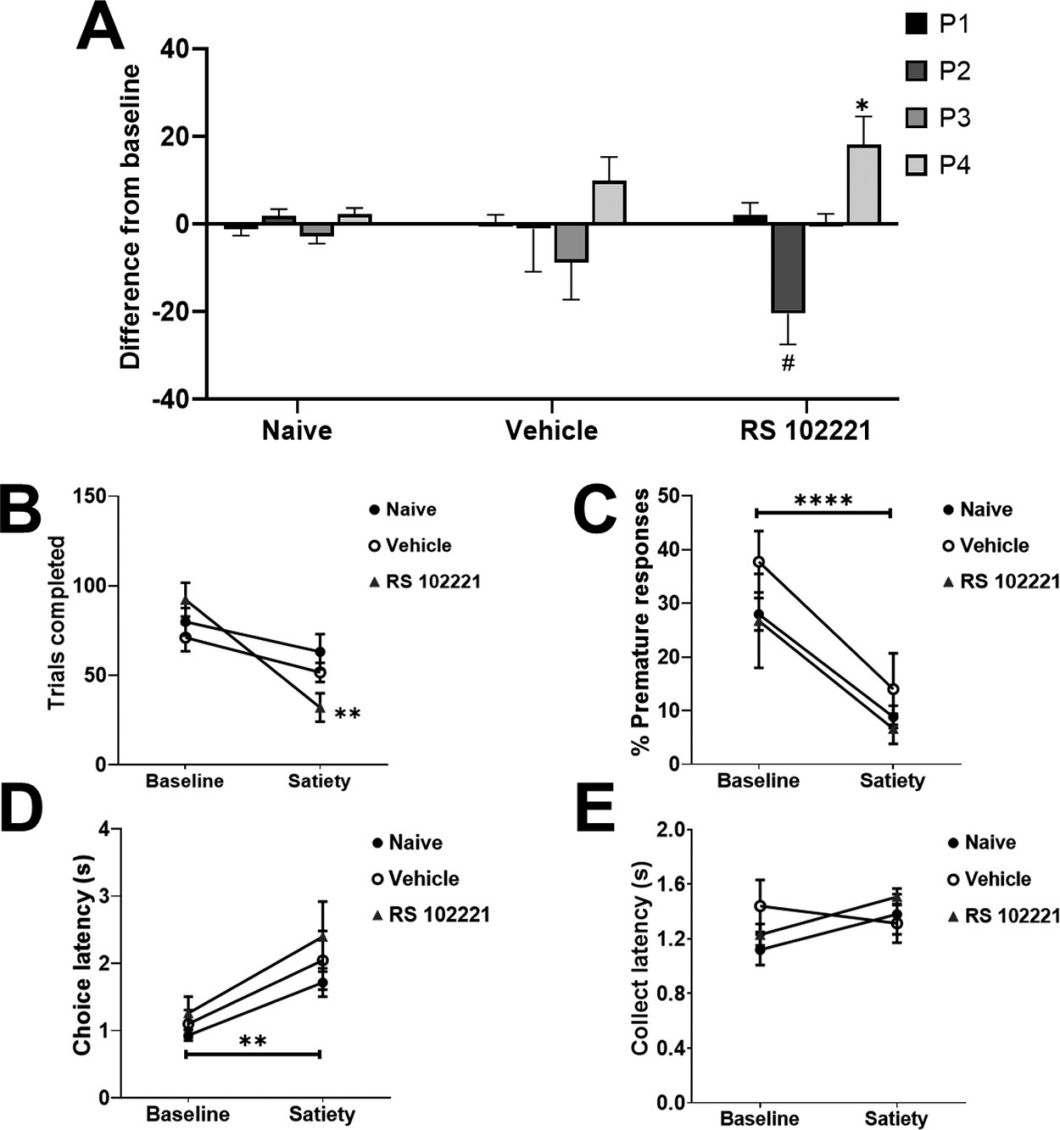
Effects of sucrose pellet devaluation on choice preference and other task variables. Only rats receiving intra-lOFC RS 102221 shifted their choice profile (***A***) and completed significantly fewer trials (***B***) in response to devaluation. Animals in all groups exhibited a significant reduction in premature responding (***C***) and increased choice latencies (***D***). No shift in latencies to collect reward were observed in any group (***E***). In panel ***A***, data are expressed as the mean change in % choice from baseline ± SEM to illustrate effects independent of differences in preference for each option between cohorts. Data are expressed as mean ± SEM in all other panels; *p < 0.05, **p < 0.01, ****p < 0.0001, #*p* = 0.07 compared with each group’s own baseline.

Behavioral data from devaluation sessions for each group of rats (naive, vehicle, or drug) was compared with their baseline data (naive group: no manipulation; vehicle group: vehicle dosing data; drug group: highest RS 102221 dose data). In Figure 5*A*, choice of the P1–P4 options is depicted as a difference in % choice between baseline and devaluation sessions (baseline subtracted from devaluation) for each group. This was done to highlight shifts in choice separate from overall cohort differences in the selection of the different options. Mean values and SEMs for each session and group can be found in Table 2. Risky and optimal rats are grouped together as statistical analyses did not reveal any effects that were dependent on risk status. We observed a significant shift in P1–P4 choice in response to reinforcer devaluation that was dependent on group but not risk status (devaluation × choice × group: *F*_(9,57)_ = 2.56, *p *=* *0.02; Fig. 5*A*). Naive rats did not demonstrate a shift in their choice profile (devaluation × choice: *F*_(3,36)_ = 1.58, *p *=* *0.21). Similarly, we did not observe any change in the choice profile of rats that received a vehicle dose in addition to devaluation, versus vehicle alone (devaluation × choice: *F*_(3,9)_ = 1.39, *p *=* *0.31). Conversely, rats in the drug + devaluation condition exhibited a shift in their decision-making that was significantly different from the effect of the drug alone (devaluation × choice: *F*_(3,12)_ = 5.64, *p *=* *0.01). When comparing P1–P4 choice between the drug versus drug + devaluation condition with *post hoc* analyses, we observed a trending reduction in P2 choice (*t*_(5)_ = 2.35, *p *=* *0.07) and a significant increase in P4 choice (*t*_(5)_ = −3.76, *p *=* *0.01). A comparison of the drug + devaluation versus vehicle condition in these rats reached marginal significance (devaluation × choice: *F*_(2,6)_ = 3.80, *p *=* *0.08), resulting from a significant increase in P1 and P4 choice (P1: *t*_(5)_ = −4.92, *p *=* *0.004; P4: *t*_(5)_ = −5.95, *p *=* *0.002). Thus, rats who received an infusion of the highest dose of RS 102221 into the lOFC were uniquely sensitive to the effects of reinforcer devaluation on choice, exhibiting a choice profile that differed from their decision-making patterns after receiving either vehicle or drug alone.

**Table 2:** Percent choice of the different options in response to acute sucrose pellet devaluation in surgically-naive rats, rats who received intra-lOFC RS 102221 in addition to devaluation, and rats who received intra-lOFC vehicle and devaluation

Group	Condition	P1	P2	P3	P4
Surgically naive	Baseline	4.82 ± 1.33	51.72 ± 9.17	24.93 ± 8.68	18.53 ± 7.18
	Devaluation	3.62 ± 1.33	53.50 ± 9.25	22.09 ± 8.12	20.79 ± 6.66
Drug + devaluation	Vehicle	1.59 ± 0.79	56.57 ± 7.93	25.91 ± 11.55	15.93 ± 8.51
	Drug	7.13 ± 1.67	58.16 ± 5.44	18.67 ± 8.12	16.04 ± 6.56
	Devaluation	9.29 ± 1.73	37.67 ± 10.06	18.94 ± 8.34	34.10 ± 12.09
Vehicle + devaluation	Vehicle	4.83 ± 2.46	41.01 ± 13.12	40.03 ± 19.23	14.13 ± 5.56
	Devaluation	4.92 ± 1.72	39.91 ± 10.92	31.19 ± 17.17	23.98 ± 9.85

Data are mean ± SEM.

#### Other task variables

We observed a significant shift in trials completed that was dependent on group (group × devaluation: *F*_(3,19)_ = 6.25, *p *=* *0.004; Fig. 5*B*). Only rats that received intra-lOFC RS 102221 completed significantly fewer trials in response to devaluation (drug: *F*_(1,4)_ = 24.12, *p *=* *0.008; vehicle: *F*_(1,3)_ = 4.64, *p *=* *0.12; naive: *F*_(1,14)_ = 2.50, *p *=* *0.14). Rats in all groups exhibited decreased premature responding (*F*_(1,19)_ = 39.37, *p *<* *0.0001; Fig. 5*C*) and increased latencies to choose an option (devaluation: *F*_(1,19)_ = 17.52, *p *=* *0.001; Fig. 5*D*) in response to reinforcer devaluation. No effect was observed on omissions or latencies to collect reward (all *F *<* *1.16, all *p *>* *0.30; Fig. 5*E*). See Table 3 for the mean values and SEMs for the reported variables in each group and session.

**Table 3:** Mean values of all other task variables (% premature response, choice latency, collect latency, omissions, trials completed) in response to acute sucrose pellet devaluation in surgically-naive rats, rats who received intra-lOFC RS 102221 in addition to devaluation, and rats who received intra-lOFC vehicle and devaluation

Group	Condition	% Premature responses	Choice latency	Collect latency	Omissions	Trials completed
Surgically naive	Baseline	27.96 ± 3.00	0.93 ± 0.08	1.12 ± 0.11	0.25 ± 0.11	79.89 ± 7.63
	Devaluation	8.82 ± 2.09	1.71 ± 0.21	1.38 ± 0.15	1.31 ± 0.55	63.14 ± 9.87
Drug + devaluation	Vehicle	26.72 ± 5.72	1.35 ± 1.20	1.57 ± 0.35	1.50 ± 1.11	80.87 ± 11.71
	Drug	19.96 ± 6.94	1.26 ± 0.25	1.23 ± 0.08	0.50 ± 0.34	92.18 ± 9.46
	Devaluation	6.61 ± 2.81	2.40 ± 0.52	1.51 ± 0.06	1.33 ± 0.49	32.00 ± 7.93
Vehicle + devaluation	Vehicle	37.74 ± 5.72	1.10 ± 0.21	1.33 ± 0.19	1.20 ± 0.97	71.06 ± 7.63
	Devaluation	14.00 ± 6.68	2.04 ± 0.43	1.31 ± 0.14	0.80 ± 0.37	51.62 ± 5.40

Data are mean ± SEM.

### Microinfusions into PrL region

#### Choice

**Figure 6.**
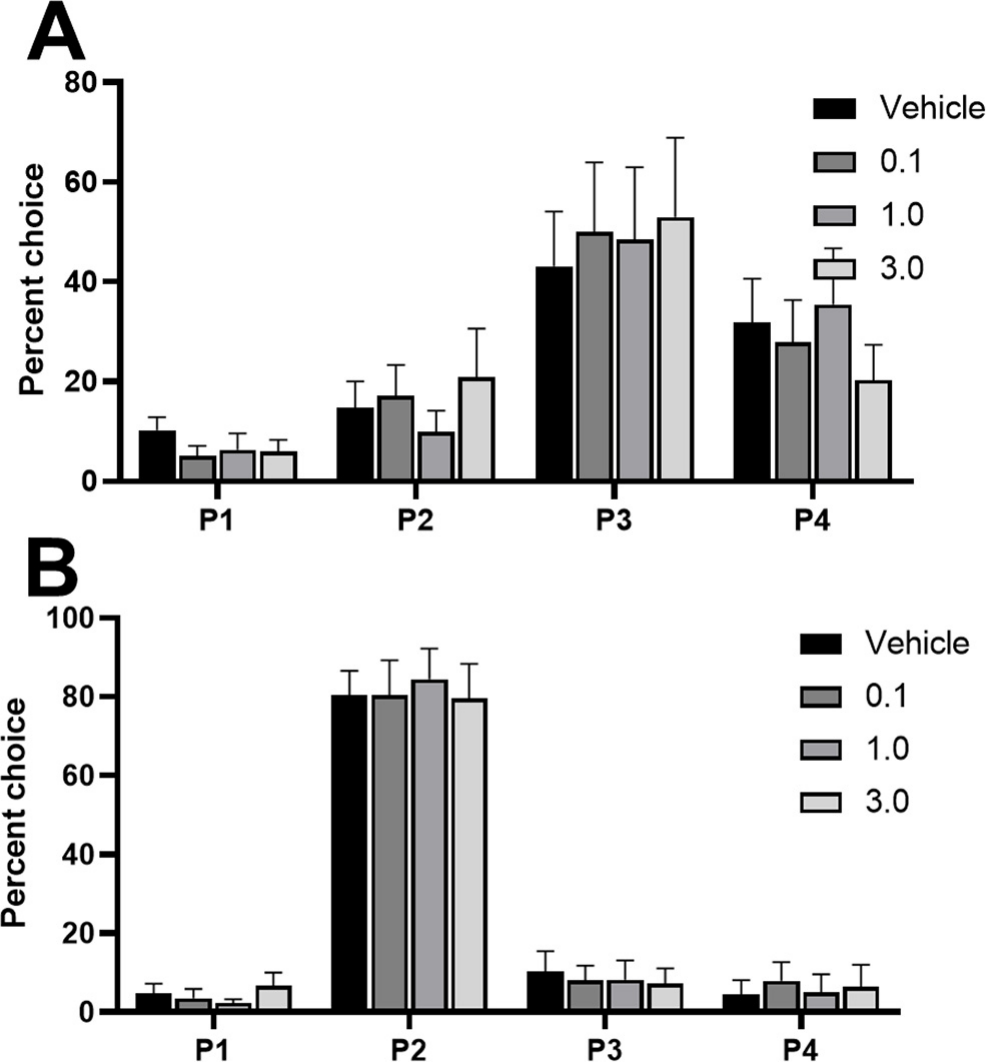
Effects of intra-PrL infusions of the 5-HT_2C_ receptor agonist RS 102221 in (***A***) risk-preferring animals (*n *=* *5) and (***B***) optimal decision-makers (*n *=* *6) on P1–P4 choice. Antagonism of the 5-HT_2C_ receptor in the PrL did not significantly increase or decrease choice of any particular option in either risk-preferring (***A***) or optimal (***B***) rats. Data are expressed as mean ± SEM.

When examining choice in an omnibus ANOVA, we observed a significant effect of dose that did not interact with risk status or the different options (dose: *F*_(3,27)_ = 3.39, *p *=* *0.03), potentially indicative of increased variability or noise in rats’ response patterns that does not reliably load on one option or another. Figure 6*A*,*B* depicts the % choice of each option at each dose in risk-preferring and optimal rats, respectively. Comparing the choice of P1–P4 between vehicle and each dose did not reveal any significant effects (all *t *<* *1.99, all *p *>* *0.09). Correspondingly, there was no significant effect on risk score (dose: *F*_(3,27)_ = 0.64, *p *=* *0.60). These results indicate that while rats’ decision-making patterns became more variable across doses, there was no clear pattern of an increase or decrease of choice for any particular option.

### Premature responding and other variables

No significant effect was observed on premature responding, latency variables, omissions, or trials completed (all *F *<* *1.68; *p *>* *0.20; see Table 1 for mean values and SEMs of task variables at each dose).

## Discussion

Results from this study demonstrated that infusing a 5-HT_2C_ antagonist directly into the lOFC, but not the PrL region, improved decision-making in risk-preferring rats without negatively impacting impulsivity measures. 5-HT_2C_ antagonism in the lOFC also restored behavioral sensitivity to reinforcer devaluation, indicating that flexibility in reward valuation was increased by this manipulation. In contrast, intra-PrL infusions of RS 102221 did not increase optimal choice. The ability of 5-HT_2C_ antagonism to ameliorate cue-enhanced risky choice therefore exhibits at least some regional specificity within the frontal cortices.

While the damage associated with surgeries and infusions is typical of studies using this technique, we cannot rule out that this may have altered the functioning of prefrontal circuitry and influenced the observed results. Nevertheless, within-subjects comparisons to vehicle dosing, along with counterbalanced orders of doses among the rats, supports the conclusion that the effect of 5-HT_2C_ antagonism in the lOFC is not simply because of procedure-associated damage. Furthermore, choice patterns during baseline sessions did not shift throughout the infusion procedure and were not significantly different from data collected before surgery.

Although the ratio of 5-HT_2A_ to 5-HT_2C_ receptors in the mPFC is positively correlated with levels of premature responding on a simplified version of the 5CSRT (Anastasio et al., 2015), we did not see any increase in premature responding when RS 102221 was delivered into the PrL, similar to a previous report (Robinson et al., 2008). There were fewer rats in the PrL cohort (*n *=* *11) than the lOFC cohort (*n *=* *18), but the sample size for both experiments was certainly comparable to other studies using this technique. Considerable data suggest an association between 5-HT_2C_ receptor activity in the mPFC, motor impulsivity, and relapse-like cocaine-seeking in rats (Filip and Cunningham, 2003; Anastasio et al., 2014; Swinford-Jackson et al., 2016). Furthermore, increased risky decision-making following cocaine self-administration correlates with at least one measure of relapse vulnerability (Ferland and Winstanley, 2017). Given that both greater risky choice and higher levels of premature responding are associated with behavioral markers of cocaine addiction in rats and are significantly correlated at the population level (Barrus et al., 2015), we might expect a greater overlap in the neurobiological control of these cognitive processes. However, numerous studies now show that these phenomena are subject to differential neural and pharmacological regulation (Zeeb et al., 2009; Barrus and Winstanley, 2016; Adams et al., 2017; Betts et al., 2021; Chernoff et al., 2021). While impulsive action and risky decision-making may interact synergistically in the manifestation of impulse control and addiction disorders, they ultimately may represent somewhat dissociable pathways to addiction.

In contrast to the effects of systemic administration, which impacted both optimal and risk-preferring rats, intra-lOFC administration of a 5-HT_2C_ antagonist only improved decision-making in those that exhibited a preference for the risky options at baseline. The neural locus whereby 5-HT_2C_ antagonism further optimizes choice in animals already making advantageous decisions remains to be determined, but is clearly neither the lOFC nor the PrL. The selective effects of drug infusions in risk-preferring rats also suggests that the neural architecture underlying the decision-making process in optimal versus risky rats differs, either in the regions forming the network, the weight given to the output of those regions in guiding choice, or the computational analyses performed by key nodes. Indeed, data across species support the view that individual differences in choice preference can be attributed to differential activity across brain networks. Cues present in the environment can also influence the adoption of a behavioral strategy; the considerable literature on sign-trackers versus goal-trackers may best exemplify the significant individual differences in how such cues can be used to guide behavior (Flagel et al., 2009, 2011; Saunders and Robinson, 2010, 2013). Illuminating a cue light during lengthy delays between response and reward delivery can decrease delay discounting (i.e., reduction in a reward’s subjective value because of waiting period) in rats (Cardinal et al., 2000). However, the presence of this cue does not eliminate the large individual differences in animals’ preferences for smaller-sooner versus larger-later rewards. Inactivation of the lOFC only reduced choice of the larger-later reward in rats that showed a high baseline preference for this option; an effect that was also observed following local infusions of dopamine antagonists (Zeeb et al., 2010). Thus, the hypothesis that recruitment of the lOFC into the decision-making process depends on the presence of cues, and that decision-making patterns are only lOFC-dependent in a subpopulation of individuals that use those cues to guide behavior, has some precedent.

It is interesting to note that the effect in risk-preferring animals was specific to a decrease in P3 (their preferred risky option) and an increase in P1, the optimal option offering the most frequent wins but the smallest reward size (one sucrose pellet). This could be because of either an increased sensitivity to the length and/or frequency of time-out penalties, or increased impact of frequent winning trials. This may help explain the specific effect observed in risk-preferring animals; for optimal rats, the difference between P1 and P2 in time-out penalty length/frequency and reward frequency may not be large enough to shift decision-making patterns away from their preferred option. Investigating neural activity following rewards and time-out penalties on each option within the lOFC could shed light on these hypotheses.

Indeed, identifying how audiovisual cues modulate lOFC neuronal firing as well as the impact of 5-HT_2C_ antagonism are important next steps. The 5-HT_2C_ receptor is an excitatory GPCR found throughout the rat central nervous system (Clemett et al., 2000). Previous studies have demonstrated that 5-HT_2C_ receptors are primarily located in the deep layers of the PFC in rats (Pompeiano et al., 1994; Liu et al., 2007). In the mPFC, at least 50% of receptors are localized to GABAergic interneurons, and are hypothesized to regulate the output of pyramidal cells (Liu et al., 2007). Considerably less is known about the localization and function of 5-HT_2C_ receptors in the OFC. Interneurons in this region play a key role in reversal learning, so it is possible that modulation of GABAergic interneuron activity by the 5-HT_2C_ antagonist may drive the improvement in choice seen here (Bissonette et al., 2015).

As noted in the introduction, previous literature has strongly implicated the 5-HT_2C_ receptor in cue-mediated behaviors such as cue-induced cocaine seeking and responding for a conditioned reinforcer through its regulation of the mesolimbic dopamine system (Pentkowski et al., 2010; Browne et al., 2017). Based on this work, 5-HT_2C_ receptor antagonists should increase motor impulsivity and potentiate the risk-promoting effect of cues in the rGT. While the former is true, the latter is clearly not. 5-HT_2C_ receptor antagonism must therefore alter decision-making through an alternate, yet concurrent mechanism. The current data implicate the lOFC as one critical locus, and an increase in behavioral flexibility as a potential cognitive mechanism, through which decision-making may improve. Serotonergic activity within the OFC, and the 2C receptor, have been implicated in cognitive flexibility by multiple previous studies (Boulougouris and Robbins, 2010; Alsiö et al., 2015, 2021; Barlow et al., 2015). Previous results have indicated that circuitry between the OFC and basolateral amygdala (BLA) supports shifts in choice following reinforcer devaluation on the uncued rGT (Zeeb and Winstanley, 2013). It may be that this circuitry is also involved in cue-induced inflexibility on the task. This pathway certainly plays a role in cue-based decision-making, as BLA-OFC projections are essential for guiding decision-making based on cue-triggered reward representations (Lichtenberg et al., 2017).

It is notable that decision-making on the cued task was not altered by reinforcer devaluation in naive or vehicle-treated rats, in contrast to the effects of this manipulation reported previously in the absence of the cues (Zeeb and Winstanley, 2013). Decision-making in other tasks that require considerably more sessions to train remain sensitive to changes in outcome value, indicating that simple repetition of actions in complex cognitive tasks is not sufficient to produce habitual behavior through procedural motor learning (Cocker et al., 2012). Indeed, goal-directed control can be maintained following prolonged training even if automatization of certain action sequences occurs (Garr and Delamater, 2019). Acute satiety with regular chow did not shift choice patterns on the uncued rGT and thus the effect of reinforcer devaluation on choice can be attributed to shifts in goal-directed action rather than reduced motivation. While acute satiety with chow has not been tested on the crGT, it stands to reason that reduced motivation alone would similarly leave choice patterns unaffected. As such, decision-making on the crGT may fail one of the critical tests of true goal-directed behavior, in that it is insensitive to changes in the goal’s value (Balleine and Dickinson, 1992). If this is the case, then 5-HT_2C_ antagonism in the lOFC may shift rats toward a goal-directed response strategy and therefore restore sensitivity to reinforcer value.

Regardless of experimental condition, motivation to engage in the task declined; premature responses decreased when animals were sated, while the latency to choose an option increased. Interestingly, while latency to collect reward is sensitive to satiety on the uncued rGT (Zeeb and Winstanley, 2013), this measure did not significantly increase with reinforcer devaluation in any group. Furthermore, the inclusion of cues on the rGT results in decreased collection latencies, particularly in risky rats (Hathaway et al., 2021). It therefore may be that the presence of the cues invigorates responding to reward delivery, and the effect is not dependent on the value of the reward or computation within the lOFC, as this measure was unaffected by devaluation with or without drug administration. In addition, the number of trials completed decreased in all rats in response to devaluation, but the effect only reached significance in rats who also received RS 102221. On the uncued rGT, trial completion was reduced in response to devaluation, but this was prevented by disconnecting the BLA and lOFC (Zeeb and Winstanley, 2013). Thus, the lOFC may play a key role in inhibiting perseverative responding on the rGT. That intra-lOFC infusion of RS 102221 selectively rendered decision-making and trial completion more sensitive to reinforcer devaluation, without altering the effects of satiety on other variables, further supports the hypothesis that local 5-HT_2C_ antagonism facilitated some form of cognitive flexibility.

As alluded to above, if changes to the value of the reinforcer do not alter behavior, that behavior is thought to be under habitual rather than goal-directed control. However, it could be that the behavior in question is instead reinforced by another aspect of the environment. Given that the cues are concurrent with reward delivery, it could be argued that “reward + cue” has formed a compound reinforcer. Selective devaluation of only one component of this reinforcer may therefore fail to alter response patterns. Certainly, in the drug addiction literature, cues that are present when drug is taken acquire incentive motivational salience that does not decline even when users do not obtain pleasure from drug ingestion (Robinson and Berridge, 1993). However, this is believed to be a highly aberrant state, driven by supraphysiological drug-induced dopamine release amplifying associative learning between cues and drugs. Why sound and light cues should exert the same effect in the current task is unclear, given that rats typically show a dramatic reduction in responding for reward-paired cues following devaluation of that reward (Hatfield et al., 1996; Pickens et al., 2005; McDannald et al., 2014). This is not the first time that similarities have been observed between responding on the crGT and responding for addictive drugs, as there is some evidence that risky choice on-task and cocaine self-administration may cross-sensitize and/or substitute for one another (Ferland et al., 2019; Hynes et al., 2021). However, systemic 5-HT_2C_ antagonism increases responding for drug (Fletcher et al., 2002), yet decreases risky choice here, indicating that the pharmacological regulation of these processes is not uniform.

Instead of the cues acquiring incentive motivational properties that are now independent of reward value, another alternative is that these highly salient audiovisual cues overshadow sucrose pellet delivery to some extent (Pavlov, 1927), such that a change in the sucrose pellet value does not dominate behavior in the presence of the cues. To our knowledge, neither the lOFC nor 5-HT_2C_ receptor signaling have been evaluated in overshadowing experiments, and as such further discussion of this hypothesis is premature. If overshadowing is taking place, then the attenuation of learning about the devalued state of the reward is highly specific to the decision-making process, as devaluation still impacted latencies and motor impulsivity, indicating a reduction in the ability of the sucrose pellet rewards to motivate and invigorate behavior.

Given that 5-HT_2C_ receptor antagonism can clearly increase incentive motivation for rewards and reward-paired cues, likely through actions in the mesolimbic dopamine pathway, the following question remains: why does this mechanism not dominate the decision-making process? One potential answer comes from human literature examining the impact of serotonin depletion on model-based behavior: goal-directed choice is impaired when learning from rewards but enhanced when learning from punishments (Worbe et al., 2016). Computational modeling analyses have revealed that the addition of cues to the rGT specifically impairs learning from the time-out penalties (Langdon et al., 2019). As such, manipulating serotonergic activity within the lOFC may promote the correct integration of punishments into the stored action-outcome contingencies for risk-preferring rats, rather than influencing the incentive motivation of the cues. This is in line with the finding that responding for a conditioned reinforcer does not correlate with risky choice on the crGT, at least in female rats (Winstanley and Tremblay, 2016), indicating that incentive motivation is not primarily responsible for cue-induced risky choice on this task. The activity of striatal neurons, although influenced by neuromodulators like dopamine, is still primarily driven by cortical inputs. It is therefore possible that 5-HT_2C_-receptor mediated modulation of lOFC output is sufficient to dominate the behavioral response.

Overall, these results indicate that 5-HT_2C_ antagonism in the lOFC can ameliorate cue-induced disadvantageous risky choice in rats with preexisting preferences for these risky options. The lack of effect in optimal rats, together with recent computational modeling analyses, suggests there are underlying differences in the processing or storage of action-outcome contingencies between optimal and risk-preferring animals. It is currently unknown whether this is because of distinct activity patterns within the lOFC or differential involvement of downstream targets. Future studies could examine activity in the lOFC during crGT learning and performance in risk-preferring versus optimal rats to address this question.

Furthermore, these results suggest that targeting flexibility may be a viable approach to improving decision-making in individuals with impaired cost/benefit decision-making, specifically in the presence of cues. This would have implications for the treatment of behavioral addictions and substance use disorders, in which individuals show marked impairments in disadvantageous risky decision-making and processing of reward-associated cues (Goudriaan et al., 2005; Limbrick-Oldfield et al., 2017; Zilberman et al., 2019). Recent clinical trials for the 5-HT_2C_ agonist in the treatment of substance use disorder have been unsuccessful. Results from these studies indicate the necessity to attend to individual differences, as only rats with preexisting deficits in decision-making show improvements in response to the antagonist. Greater specificity of targeting to regions may also improve treatment, as the antagonist can increase impulsivity via other pathways. Allosteric modulators of the 5-HT_2C_ receptor may be worth pursuing in this regard. Given that numerous effective psychoactive medications act on the serotonin system, there is every reason to feel cautiously optimistic that a viable serotonergic medication could be developed for disorders hallmarked or exacerbated by risky decision-making.

## Synthesis

Reviewing Editor: Jonathan Lee, University of Birmingham Decisions are customarily a result of the Reviewing Editor and the peer reviewers coming together and discussing their recommendations until a consensus is reached. When revisions are invited, a fact-based synthesis statement explaining their decision and outlining what is needed to prepare a revision will be listed below. The following reviewer(s) agreed to reveal their identity: Laura Bradfield, Emma Robinson.

The reviewers and reviewing editor were in agreement that the manuscript presents a logical extension of previous work in the field, integrating interesting insights into the function of serotonin in the lOFC. It is well designed and appropriately analysed, and largely reasonably interpreted.

The full reviews are appended below, but I wanted to highlight two points that warrant particular attention in any revision of the manuscript.

First, the presentation and interpretation of the “devaluation” experiment could be clearer and more carefully described. We acknowledge that there may be differences in the precise interpretation of certain terms within the field, but the use of the term “devaluation", when describing an experiment that involves pre-feeding the original outcome risks confusion in relation to general motivational effects vs true devaluation of the specific outcome (i.e. sensory-specific satiety). Please see the detailed comments from Reviewer 1.

Second, and somewhat related again to the devaluation experiment, but also more widely applicable to other areas of the results, the text could more sympathetically guide the reader through the various panels of the figures in order more clearly to elucidate how specific data support particular statements (please see Reviewer 1 comments for further details). One salient example of this is the data in figures 4 & 5 and their different modes of presentation that has confused us (see Reviewer 2 comments).

-----------------------------------------------

<i>Reviewer 1 comments</i>

In this manuscript, the authors investigate the roles of serotonin receptors in the lateral orbitofrontal cortex (lOFC) and prelimbic cortex (PrL) in risky decision-making using the rat gambling task - a version of the Iowa gambling task. They found that intra-lOFC infusions of the 5-HT2c receptor antagonist RS 102221 reduced risky choice in risk-preferring rats, but did not affect decisions in optimal decision-makers. Similar infusions in the PrL did not have the same effect, but apparently did have a blanket dose-dependent effect on responding. The authors also explored the effects of satiation on responding, but this only reduced overall responding as far as I can tell, without affecting the pattern of responding. The authors conclude that 5-HT2c receptors in the lOFC promote risky choice patterns.

Overall I think this is a nice paper, although perhaps only moderately novel and exciting. It is nicely written, with some interesting insights into serotonin function in lOFC the introduction and discussion. The authors have clearly thought through their work thoroughly. I do have a few issues and questions that should be addressed, which I will outline below.

Major

1. The ‘devaluation’ procedures used here lack the proper controls, and therefore cannot be used to infer anything about goal-directed action. That is, devaluation of a reward via sensory-specific satiety requires a control that is sated on a different outcome to that which is used in the task (hence the term ‘sensory-specific’ satiety). Without this control, the authors are both devaluing the outcome and altering the motivational state from hungry to sated in the ‘devalued’ animals, and either of these factors could influence responding. I would contend that reduced motivation is in fact more consistent with the observed results of this manipulation, which appears to have altered task engagement without altering the response profile/pattern of responding. An alteration in goal-directed action would, I think, be more likely to differentially affect the selection of optimal vs. risky options. I suggest that the authors alter their language throughout the manuscript to reflect the fact that these could be devaluation effects, or could simply be motivational, and that the latter is more likely given the general reduction in task engagement.

2. The way in which the results were reported I found quite confusing to follow. I would prefer it if each effect was referred to a specific graph, or particular part of a graph, rather than a blanket statement at the end saying “see Figure X for a summary of differences” (for example). That is, if there is an effect in risk-preferring rats, and these data are shown in the top panel of the figure, could the text please refer to the top panel of the figure? If it could be made clearer which reported effects and statistics relate to which graph/part of a graph, this would be much easier to follow.

3. The PrL data seem like a bit of an afterthought. For example, there’s a lot of introduction to why they would infuse into the lOFC, but almost no rationale given for the PrL infusions. Could the authors insert some more rationale for this, as it’s quite jarring when it is mentioned? I also found the results for Figure 6 difficult to understand. The authors say there is an effect of dose that did not interact with any other variable. Do they mean that general responding increased as dose increased? Across all rats? This would suggest the pattern stayed the same (in contrast to what is claimed) although I’m not sure it is consistent with what I see in Figure 6. It needs to be made clearer whether this is a main effect averaged across the other variables, and if so, what does it suggest? Finally, I also don’t think that ‘visual inspection’ (page 22) without statistics is sufficient to make differential claims about how decision-making was affected in risk-preferring vs. optimal decision-makers.

Minor points

4. On Page 4 it says “These findings suggest that 5-HT2c receptor antagonism enhances the control of cues over behaviour, and 5-HT2c agonism attenuates it.” Is this sentence specifically referring to antagonism/agonism in the nucleus accumbens? They need to say so otherwise the next immediate sentence appears to contradict it.

5. Page 11 - for the statistically stable baseline, when exactly did the 4 sessions occur that this number was taken from?

6. It took me some time to work out which holes in Figure 1 were P1 and P2. Initially I assumed it was the first two holes (i.e. they just went in order from P1-P4 from left to right). After some reading, I think that P1 and P2 are represented in Figure 1 as hole 1 and 3 - the ‘better options’, and P2 and P4 are holes 2 and 4 - the worse options. If this is correct, I’m not sure if there’s a way the authors could make this a bit clearer so that the reader can avoid confusion when trying to determine, for example, how the risk score is calculated?

7. It appears that risk-preferring rats did not alter their responding at P2 and P4, as a result of lOFC infusions (Figure 4A), but this is not reported on. I think it should be mentioned. Why might lOFC infusions selectively affect responding at P1 and P3?

<i>Reviewer 2 comments</i>

This study used a rat gambling task to investigate the role of 5-HT2C receptors in different sub-regions of the prefrontal cortex in risky decision-making behaviour. The authors link their findings to changes in flexible behaviour suggesting this is a neurocognitive mechanism medicating cue-enhanced risky decision-making behaviour. Overall I found the work was clearly presented and aligns with other studies of this type in terms of the technical approach and data analysis. I have a few points which I think would be useful to include to clarify some aspects of the study design and task and I also have a couple of general points about the data analysis and interpretation. I want to emphasise that the more general points are to me important to be discussed but are not things that I see as fundamental to the quality of the study as presented if that makes sense.

In the description of the food restriction, 14g per animal seems quite low, could the authors clarify if animals were managed to 85% free feeding weight match to normal growth or maintained at 85% of their initial weight? Could the authors provide weight data for the animals at the start of the pharmacological studies?

What environmental enrichment was provided and if none, please state this and explain why.

I was not quite clear from the description of the training exactly how long the rats took to acquire the task before surgery and testing, perhaps this could be added to the methods section. More generally, if the animals are requiring multiple sessions to learn the different contingencies, how do the authors resolve this against the concepts that the behaviour is flexible and dynamic enough to be sensitive to acute drug effects and decision-making? I am not sure if this has been tested previously but if the rules for each of the locations was changed, how long would the rats take to adapt their strategy?

The n numbers of the different sub-groups do seem low for this type of cognitive readout although I acknowledge that this is not an unusual n number to see reported for this type of operant method. If you consider the effect size expected for a clinical scenario, how do you relate this to what would seem to be a very large effect in these animals?

There does appear to be quite extensive damage associated with the infusion site and more than I would have expected to see given the methods described. I wonder if the authors might comment on this and the potential impacts on the prefrontal circuitry?

The authors have provided data from a study where they use a satiety test, but I am not sure I fully followed the interpretation of the subsequent dataset. The primary outcome in figure 4 is shown by group but the data in figure 5 is presented differently. If the aim is to relate the effects, then I am not sure why the data are presented differently? Also, in the discussion the focus is on goal-directed versus habitual behaviour but what about procedural learning which can also affect how rats perform these tasks with long training schedules.

Paragraph 2 of the discussion, n=12 versus n=11 earlier for prelimbic.
